# Metal-organic framework (MOF)-bioactive glass (BG) systems for biomedical applications - A review

**DOI:** 10.1016/j.mtbio.2024.101413

**Published:** 2024-12-18

**Authors:** Andrada-Ioana Damian-Buda, Nariman Alipanah, Faina Bider, Orhan Sisman, Zuzana Neščáková, Aldo R. Boccaccini

**Affiliations:** a Institute of Biomaterials, Department of Materials Science and Engineering, University of Erlangen–Nuremberg, Cauerstraße 6, 91058, Erlangen, Germany; b FunGlass – Centre for Functional and Surface Functionalized Glass, Alexander Dubček University of Trenčín, 911 50, Trenčín, Slovakia

**Keywords:** Metal-organic-framework, Bioactive glass, Tissue engineering, Biomedical applications

## Abstract

In recent years, metal-organic frameworks (MOFs) have emerged as promising materials for biomedical applications, owing to their superior chemical versatility, unique textural properties and enhanced mechanical properties. However, their fast and uncontrolled degradation, together with the reduced bioactivity have restricted their clinical potential. To overcome these limitations, MOFs can be synergistically combined with other materials, such as bioactive glasses (BGs), known for their bioactivity and therapeutic ion releasing capabilities. Besides comparing MOFs and BGs, this review aims to present the latest achievements of different MOFs/BGs materials, with a particular focus on their complementary and synergistic properties. Key findings show that combining MOFs and BGs enables the development of composite materials with superior physicochemical and biological properties. Moreover, by choosing appropriate processing techniques, BGs and MOFs can be fabricated as scaffolds or coatings with fast mineralization ability and high corrosion resistance. In addition, incorporation of MOFs/BGs in hydrogels improves mechanical stability, bioactivity and antibacterial properties, while maintaining biocompatibility. The mechanisms behind the antibacterial properties, likely coming from the release of metal ions and organic ligands, are also discussed. Overall, this review highlights the current research directions and emerging trends in the synergistic use of MOFs and BGs for biomedical applications, which represents a novel strategy for developing a new family of advanced therapeutic materials.

## Graphical abstract

**Figure:**
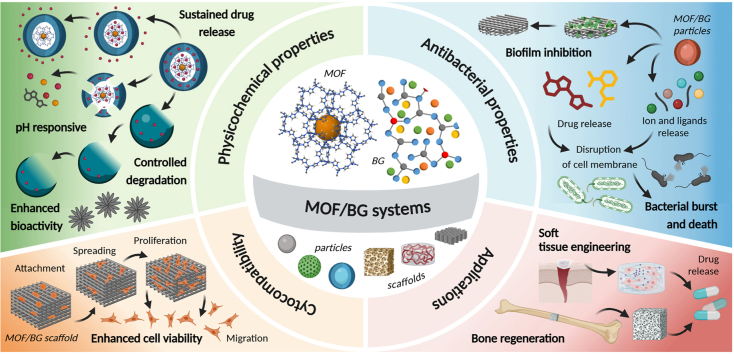

## 1 Introduction

### 1.1 MOFs in biomedical applications: general considerations

Metal-organic frameworks (MOFs) are porous materials formed by combining metal-containing nodes with organic binding ligands, resulting in an open crystalline framework with enduring porosity [[1], [2], [3]]. The wide variety of possible ligands and metal-containing units has led to more than 20,000 different types of MOFs with tunable geometry, surface area, size, and pore functionality. These properties make them suitable for different applications, ranging from catalysis to drug delivery systems [[1], [4]]. In the context of the medical field, both the type of metal nodes and organic linkers can be decisive in determining the biocompatibility of MOFs. Metal nodes can influence the biocompatibility either through the generation of reactive oxygen species (ROS) or by their ability to coordinate different biomolecules [[5], [6]] On the other hand, for the organic linkers, the different hydrophobic-hydrophilic balance (log P) mechanisms are thought to influence the interaction of MOFs with biological systems [[5], [6]].

The high loading capacity, tunable composition, high degradability and controlled release of MOFs make them suitable for biomedical imaging, coatings, biosensors, cancer treatment and theranostic devices [[3], [4], [7], [8], [9]]. Furthermore, MOFs can be manipulated to be positively or negatively charged, allowing the incorporation of different biomolecules or metal ions [9]. In this way, MOFs can attain strong antiviral, antibacterial and antifungal properties. For instance, Jaros et al. [10] successfully synthesized Ag-containing MOFs by assembling silver oxide, 1,3,5-triaza-7-phosphaadamantane (PTA), and pyromellitic acid (H_4_pma), which exhibited strong antibacterial and antiviral properties, most likely due to the compounds released from the MOF structure. To further expand the range of available compositions of MOFs to core-shell or well-mixed structures, researchers have recently focused on using post-synthetic exchange method (PSE), also known as ‘solvent-assisted linker exchange’ method (SALE). This technique relies on the exchange of the original linker in the initial MOF structure (parent MOF) with another linker (daughter linker). Importantly, this process does not change the topology of the parent MOF, but it significantly broadens the structural and functional properties of MOFs [[11], [12], [13]]. While comprehensive reviews on MOFs science and technology are available [[14], [15], [16]], this review specifically focuses on the types of MOFs designed and applied in the biomedical field, in particular combined with bioactive glasses, as introduced below.

Among different biomedical applications, MOFs are mostly studied as controlled drug delivery systems, with zeolitic imidazolate frameworks (ZIFs) being chosen for their acid-responsive degradability [17]. Hence, ZIFs show great potential as pH-responsive drug carriers, particularly suited for the targeted delivery of antibacterial agents [18]. Drug loading into MOFs is usually achieved via wet impregnation, a step which comes subsequently after MOF activation [[19], [20]]. For example, di Nunzio et al. [21] proposed an efficient “ship-in-a-bottle” strategy by directly encapsulating one monomer of topotecan (TPT, 1) within the pores of biodegradable MIL-100 Nano-MOF. In another approach, proposed by Bim-Júnior et al. [22], a one-step water-based method was employed to encapsulate quercetin (QCT) into zeolitic imidazolate framework-8 (ZIF-8) during MOF synthesis (QCT@ZIF-8). Even though the particle size of the QCT@ZIF-8 slightly decreased, they maintained their initial cuboidal morphology and crystalline structure, while achieving a high encapsulation efficiency of over 89 %. The drug release study conducted at pH = 7.4 unveiled an initial burst release of 36 %, followed by a sustained release for up to 240 h. In contrast, under acidic conditions (pH = 5.2), QCT was released completely within less than 60 min, which might have been caused by the breakage of the coordination bonds between Zn^2+^ and imidazole rings. Given the presence of acidogenic bacteria in the oral environment, QCT@ZIF-8 particles were further incorporated into a dental resin. Under these acidic conditions, QCT can be quickly and completely released, leading to bacterial death, as supported by the results of the antibacterial assay carried out on *Streptococcus mutans* bacterial strain [22].

In a similar work, reported by Ali et al. [23], MOF-based drug delivery systems with strong antibacterial properties against *E. coli* and *S. aureus* were developed. More precisely, gentamicin-loaded Zn-containing MOFs were incorporated into oxidized chitosan matrix to tackle bacterial infection. After confirming the presence of gentamicin in the Zn-MOFs and subsequently in the hydrogel, antibacterial tests were performed. As expected, the composite materials showed strong antibacterial effects against strains that are more sensitive to gentamicin. Surprisingly, the materials also had antibacterial properties against strains typically resistant to gentamicin, which might come from the synergistic antibacterial effect of combining gentamicin, Zn^2+^ ions released from the MOF structure and chitosan.

If in the previously presented studies, traditional drugs were the primary focus of the investigations. Researchers have recently directed their attention towards natural-based remedies as alternatives to conventional synthetic compounds. In line with this idea, Niu et al. [24] assessed the suitability of γ-cyclodextrin-based MOFs (γ-CD-MOFs) as nanocarriers for trans-N-p-coumaroyltyramine (NCT), a natural drug isolated from *Dioscoreaceae* or *Polygonaceae* plants. The authors investigated the effect of two NCT loading methods, namely wet impregnation (NCT@CD-MOF1) and co-crystallization (NCT@CD-MOF2), on the NCT loading and release kinetics. While both techniques increased the solubility of NCT compared to the free drug, NCT@CD-MOF2 demonstrated a higher loading capacity and a more controlled drug release. This difference was attributed to the low stability of NCT@CD-MOF1 in aqueous environment, as scanning electron microscopy (SEM) analysis revealed the loss of structural stability of the CD-MOFs post-loading. Importantly, loading NCT into MOFs not only reduced its toxicity, but also maintained its inhibitory activity against α-glucosidase, demonstrating the potential of these NCT@CD-MOF systems for treating type 2 diabetes mellitus. Besides NCT, MOFs were investigated for the delivery of *Daphne mucronata* extract, which was further incorporated into electrospun polyvinyl alcohol (PVA)/polyvinyl pyrrolidone (PVP) fibers [25]. The presence of the inorganic component (MOF) ensured a significantly higher flexural and compressive strength, whereas the plant extract together with the ions released from the MOF structure imparted enhanced antibacterial, antitumoral and antifungal properties. Thus, the synergistic combination of different biomaterials with complementary properties highlights the possible application of these MOF-based PVA/PVP fibers as wound dressings.

Another critical aspect in designing a highly effective drug delivery system is to precisely target the desired tissue to maximize the therapeutic efficacy of the active compound, while reducing its systemic cytotoxicity. In this regard, Zhong et al. [26] reported the successful synthesis and characterization of a SiO_2_-MOF-Fe/Al microcontroller, an innovative approach towards the development of targeted drug delivery systems for the central nervous system. SiO_2_ particles acted as a support for covalently binding amino functionalized MIL-101(Fe), with MOF providing high drug loading and controlled release. Additionally, in order to magnetically control the movement of the microcontroller, an additional Fe/Al layer was deposited on top of the structure, which also improved its biocompatibility. To simulate the movement of the SiO_2_-MOF-Fe/Al microcontroller in the central nervous system, the researchers used an organ-on-chip device, containing an artificial cerebrospinal fluid (aCSF) to mimic the fluidic environment and a chicken embryo chorioallantoic membrane (CAM) as a model for the neural tissue. The experiment proved the ability of these SiO_2_-MOF-Fe/Al systems to follow the direction and intensity of the external magnetic field, with information about the movement mechanisms. The detailed analysis of the microcontroller's motion provides valuable information about how the drug-loading capabilities of MOFs can be integrated with stimuli-responsive materials for targeted drug delivery.

Besides drug delivery, MOFs are also investigated for theranostic approaches or coatings for bone implants [27]. As presented by Zhao et al. [27], by carefully choosing the appropriate MOF new materials with enhanced bone regeneration, antibacterial action, anti-inflammatory response, and even anti-tumoral effects can be obtained. In this regard, Fu et al. [28] investigated polydopamine modified (PDA) ZIF-8 (PZIF-8) particles as fillers for polycaprolactone (PCL) coatings on Mg alloys. The results proved the successful coating of ZIF-8 with PDA, without changing the structural integrity and crystallinity of ZIF-8. Further incorporation of PZIF-8 particles into PCL coating not only increased the surface roughness, but it also improved the bonding strength with the substrate. The PZIF-8 particles conferred to the Mg substrate higher corrosion resistance, bioactivity and improved cell adhesion, highlighting the benefits of PZIF-8 PCL composites as coatings for Mg-based orthopedic implants. Domke et al. [29] proposed a similar strategy to enhance the bioactivity and antibacterial properties of Ti6Al4V substrates by employing MOFs as biocompatible coatings. More precisely, gallic acid was used as a natural organic linker and Zn^2+^ ions as the metal component within the MOF structure. To improve the compatibility between the substrate and the coating, Ti6Al4V samples underwent an alkaline treatment with NaOH before the addition of MOF precursors. SEM images along with Fourier-transform infrared (FTIR) spectra and mapping, confirmed that the surface of the metal was covered with a uniform layer of Zn-MOFs which accelerated the mineralization process, while it also provided superior antibacterial properties compared to simple Ti substrates. In a related work, Chen et al. [30] explored the possibility of adding different bimetallic Mg/Cu-MOFs on the surface of Zn membranes, aiming to enhance their biological performance for bone regeneration. Their findings revealed that the Zn^2+^ ions released from the membrane together with the Mg^2+^ and Cu^2+^ ions coming from the MOFs had a synergistic effect on accelerating the deposition of a calcium phosphate layer, enhancing osteoblast proliferation, vascularization and antibacterial efficacy against *S. aureus* and *E. coli*.

The use of MOFs in biomedical applications includes also tissue engineering and regenerative medicine, where MOFs have been selected for enhancing cell attachment and promoting tissue regeneration. For instance, ZIF-8 nanoparticles were incorporated into the polycaprolactone (PCL) + gelatin layer of the electrospun PCL/PCL + gelatin nanofiber membrane, serving as a guiding tissue regeneration membrane for periodontal defects [31]. The slow release of Zn^2+^ from ZIF-8 nanoparticles conferred to the membrane antibacterial properties against *E. coli* and *S. aureus*, while simultaneously promoting bone tissue regeneration both in vitro and in vivo. On the other hand, MOFs, prepared by solution precipitation, mechanochemical method or solvothermal methods, possess promising properties for wound healing applications and neuronal or bone tissue engineering (BTE) [32]. For example, Shuai et al. [33] focused on the development of ZIF-8 reinforced poly-L-lactic acid (PLLA) scaffolds for BTE. Despite the high surface area and tunable porosity of ZIF-8, they undergo rapid degradation, releasing a high amount of Zn^2+^ in a short time, triggering in this way cell apoptosis [33]. To tackle this challenge, ZIF-8 particles were coated with a thin dopamine layer, which further polymerizes into PDA (ZIF-8@PDA). It was shown that this coating controls the degradation of ZIF-8, while facilitating hydroxyapatite (HAp) nucleation and accelerating the bioactivity process. PLLA scaffolds incorporating ZIF-8@PDA-HAp exhibited delayed degradation, attributed to the presence of the PDA-HAp layer, which hinders the degradation of ZIF-8 and, thus, prevents the autocatalytic degradation of PLLA. As expected, the scaffolds showed enhanced bioactivity and good cell attachment, proving the beneficial effects of using PLLA scaffolds incorporating ZIF-8 with controlled degradation provided by the PDA-HAp layer [33].

### 1.2 MOFs in combination with other biomaterials: enhanced properties

To further extend the functionalities and applications of MOFs, they can be combined with other biomaterials. For instance, Lim et al. [34] discussed in detail the possibility of incorporating MOFs in hydrogels, enabling in this way the use of MOFs-hydrogel composites in wound dressing applications. Furthermore, MOFs nanorods made of emodin sequences linked by Mg^2+^ ions were successfully added to methacryloyl/hyaluronic acid hydrogels to produce microcapsules for treating diabetic bone injuries. The presence of the MOF nanorods had a positive effect on maintaining a normal glucose metabolism, while they inhibited the inflammatory responses and minimized the oxidative stress. Simultaneously, the wound healing process and angiogenesis were stimulated both in vitro and in vivo [35]. Similar observations regarding the positive effect of MOFs-based hydrogel on regulating the diabetic bone healing process were reported by Gong et al. [36]. Compared to the work presented by Wang et al. [35], Gong et al. [36] loaded glucose oxidase in the pores of a Mg-gallic acid MOF along with the addition of another inorganic phase, HAp. This complex hybrid injectable hydrogel demonstrated angiogenic, anti-inflammatory and anti-oxidant properties which play a central role in regulating the diabetic microenvironment, thus proving the promising potential of these systems for treating diabetic wounds. The therapeutic potential of these hydrogels can be extended to antibacterial properties by encapsulating different types of MOFs (Cu-MOFs, Co-MOFs and Zn-MOFs), as shown in previous works conducted by Gwon et al. [[37], [38]].

Besides incorporating them in hydrogels, MOFs can also be integrated into thermogels without altering their rheological properties, while the pore size, geometry, and hydrophobic characteristics of MOFs facilitate the concurrent dual drug release [39]. Yang et al. [40] proposed a magnesium/gallic acid - MOF laden aerogel for skin tissue engineering, whereas hybrid systems combining MOFs with metallic nanoparticles and metal oxides/peroxides hold promise in photodynamic therapy (PDT) by mitigating tumor hypoxia [41]. For instance, Hf^4+^ – porphyrin based DBP-UiO MOFs have demonstrated high potential as photosensitizer (PS) for PDT of resistant head and neck cancer [42]. Hf^4+^ ions were selected for their strong affinity to coordinate various organic linkers and their ability to generate reactive free radicals, which could induce cancer cells apoptosis. To avoid the aggregation of Hf^4+^ ions and prevent the suppression of the excited state, Hf^4+^ ions were incorporated into a DBP-UiO network. This nanoscale core-shell MOF structure not only increases the efficacy of the photosensitizer, but also it controls the targeted diffusion of the reactive oxygen species out of the system towards the tumoral tissue. Upon light activation, the Hf^4+^ – porphyrin DBP-UiO system showed superior PDT efficacy both in vitro on SQ20B cell line and in vivo [42]. Similar enhancements of PDT were recorded for chlorin-based DBC-UiO MOFs for treating colon cancer [43]. Even though the previously presented UiO MOFs have strong anti-tumoral effects, they can be harmful for the surrounding tissue. To minimize the toxic effect on healthy cells, Zhang et al. [44] proposed a smart PDT nanoparticle system consisting of a porphyrin-based MOF core coated with MnO_2_, on which cancer cell membranes were further used as an additional shell layer (CM-MMNP). If the MnO_2_ layer provided a higher O_2_ yield in the tissue, the presence of the tumoral ligands on the surface of the MOFs was aimed to precisely target the cancer cells. The results revealed that compared to pristine MOFs, the O_2_ production of the CM-MMNP was higher in acidic tumoral environment (pH = 5.5–6.5), with a significant reduction of the probability of producing excessive O_2_ in healthy tissue (pH = 7.4) [44]. All these results highlight the pH dependent properties of the CM-MMNP that enhances the PDT and minimizes the treatment's side effects. Similar modified MOFs based nanoplatforms for PDT were recently discussed in detail by Gao et al. [45]. Furthermore, MOFs can be used in photothermal therapy (PTT), as they can achieve high tumor targeting and penetration by easily attaching large amounts of photothermite to the MOF structure. Another approach for treating cancer involves using MOFs in immunotherapy (IT) either by loading selective immune biomolecules or by including metal ions which can stimulate the immune system or modulate tumor inflammation [46]. However, the most promising results were achieved when these monotherapies (chemotherapy, PDT, PTT, and IT) were combined with other treatments (gene therapy, enzyme therapy, etc.), with MOFs enhancing the specificity and therapeutic efficacy of these treatments [46]. For example, Liu et al. [47] developed a novel nano MOF that effectively integrates radiotherapy (RT) and radiodynamic therapy (RDT). In order to combine both therapies. Hf^4+^ ions, recognized for their strong X-ray attenuation properties, were utilized together with tetrakis(4-carboxyphenyl) porphyrin (TCPP) organic linker, which also acts as a photosensitizer. The obtained MOF was further coated with a polyethylene glycol (PEG) layer to improve its biocompatibility. This advanced MOF system showed superior anti-cancer therapeutic properties in vivo, most probably as a result of the synergistic effects of the components present in the MOF system. Besides treating cancer, MOFs have emerged as novel platforms for lung cancer detection, as they can significantly improve the sensitivity of biosensors [46]. The higher sensitivity can be attributed to the wide range of metals which can be included in the MOF structure. Small changes in the concentration of cancer biomarkers in the surroundings of these MOF based biosensors can lead to small, but strong enough changes in the electronic properties of the materials which can be easily detected [46]. Despite their promising properties, further efforts should be directed towards improving the stability and scalability of such novel biosensors.

### 1.3 Bioactive glasses as suitable partner for MOFs

Other biomaterials that can be employed in combination with MOFs are bioactive glasses (BGs). BGs are inorganic materials characterized by specific silicate, phosphate or borate compositions, most commonly synthesized by melt-quenching or sol-gel methods [[48], [49]]. The properties and applications of BGs can be controlled by tailoring their chemical composition or modifying the fabrication techniques to obtain different structures (nanoparticles, fibers, 3D porous structures, etc.) [[48], [50]]. BGs stand out due to their exceptional bioactivity (bioreactivity), which is characterized by the formation of a layer of HAp on their surface when BGs are in contact with aqueous environments, such as body fluids. This bioreactivity mediates the interaction of the material with surrounding tissues [[50], [51]]. Moreover, different ions in the BG network, which can be released at controlled rates, have been shown to have therapeutic effects (angiogenesis, osteogenesis, antibacterial properties, anti-inflammatory, etc.) [52]. To further enhance the therapeutic potential of such biologically active ions, BGs can be combined with bioactive molecules or drugs, synergistically improving the biological effects of both ions and biomolecules [[53], [54]]. In this regard, mesoporous bioactive glass nanoparticles (MBGNs) with high surface area (>500 m^2^/g), large pore volume (>1 cm^3^/g), tunable porosity and narrow pore size distribution are being developed and have emerged as successful carriers for local and targeted drug release [[55], [56], [57], [58]]. For instance, Wang et al. [59] developed multifunctional core-shell nanocomposite nanoparticles using a layer-by-layer approach. In these systems, upconversion nanoparticles (UCNP) served as the core, which were first coated with a layer of SiO_2_ and then covered with mesoporous bioactive glass (MBG) layers of different compositions (UCNPs@SiO_2_@mSiO_2_-XCa (Ca/Si molar ratio 0, 5, 10, 15, and 20)). The UCNP enabled deep tissue penetration with minimal toxicity to the healthy tissue, while the shell provided controlled and sustained release of anti-cancer drugs. Furthermore, the bioactive properties of the MBGs promoted osteogenic differentiation of bone marrow stromal cells (BMSCs). To exploit both the bioactivity and tailorable composition of BGs, Oguzlar et al. [60] doped sol-gel derived silicate BGs with the rare elements ytterbium (Yb^3+^), gadolinium (Gd^3+^), and europium (Eu^3+^), known for their fluorescence and therapeutic properties. These doped BGs were then incorporated alongside meso-tetraphenyl porphyrin (H_2_TPPS) into poly(trimethylsilylpropyne) [poly(TMSP)] matrices. The BGs not only provided the system superior bioactive and therapeutic properties, but they also increased the oxygen sensitivity of the host material, making it suitable as optical oxygen sensor. Owing to their high biocompatibility and osteostimulating abilities, BGs have their main applications in hard tissue regeneration as bone scaffolds, bone substitute materials or drug carriers and coatings for implants [[48], [50], [61]] Moreover, recent studies have increasingly explored the potential of BGs in soft tissue repair, wound healing and drug delivery [[62], [63], [64]].

**Fig. 1:**
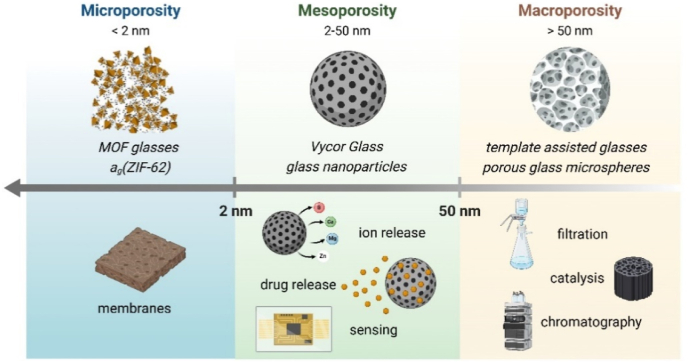
Pore size distribution range of different types of glasses and their corresponding applications. Created with BioRender.com.

In contrast to BGs, MOF glasses are relatively new and remarkable materials with unique porosity [65]. The classification of porous glasses according to the pore size range, as established by the International Union of Pure and Applied Chemistry (IUPAC), along with their fields of application, is presented in Fig. 1 [66]. The main advantage of MOF glasses compared to traditional MOFs is the absence of grain boundaries, which facilitates mass transport and gas permeation [[67], [68]]. Several studies on MOF glasses and their hybrid compositions have been published, trying to improve their mechanical properties and introduce new functionalities [[69], [70], [71], [72]]. For example, Chester et al. [73] highlighted the potential of combining MOFs and inorganic glasses to develop drug delivery systems that have bacteriological properties, with the capability of delivering chemotherapy agents. However, the properties of these MOFs - inorganic glass composites (MOFs-IGCs) can be compromised by insufficient bonding interaction between the two components. In this regard, Castillo-Blas et al. [74] proved that by applying the right heat treatment, Na_2_O-P_2_O_5_ inorganic glass can be combined with (ZIF-8) MOFs exhibiting a significant reduction of the size of the interfacial region. Such interface should ensure a smooth transition of mechanical and physical properties between the glass matrix and ZIF-8, thus enhancing the performance of the composite material. Despite improved mechanical properties, MOF glasses exhibit lower porosity compared to crystalline MOFs, which may compromise their drug loading capacities [75]. To overcome this problem, recent research has been focused on adapting the techniques used to generate porosity in inorganic glasses to MOF glasses [75].

**Fig. 2:**
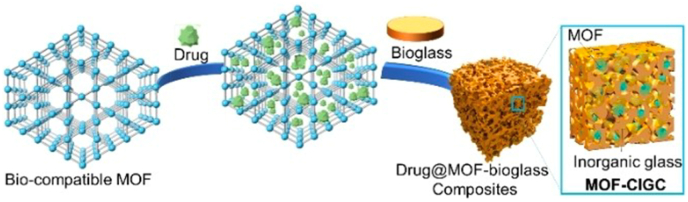
Schematic representation illustrating the incorporation of a drug within a biocompatible MOF, which is then combined with a BG to obtain a MOF-BG composite, according to Chester et al. [73]. Reproduced from Chester et al. [73] under a Creative Commons CC BY License.

Combining MOFs and BGs could lead to a new family of multifunctional composite materials suitable for drug delivery and tissue regeneration [73]. This approach emerges as a solution to overcome the challenges associated with MOF processability, while having a biocompatible support for drug delivery. For instance, Fig. 2 shows the possibility of loading a drug in a MOF, followed by mixing it with BG to create a highly controlled composite drug delivery system. On the other hand, in the context of implants, calcium-based bioactive MOFs are promising candidates for synergizing with BGs, as they can have the capacity to stimulate osteogenesis through the generation of HAp [73].

Given the advantageous use of MOFs and BGs for the development of new biomaterials, this review focuses on presenting the latest achievements in the field over the past 5 years. Therefore, this work is divided into two principal sections, i) Comparison of MOFs with BGs and ii) Synergistic incorporation of MOFs and BGs in composite systems. After this brief introduction about MOFs, MOFs and BGs will be compared to highlight the advantages and drawbacks of each material. The following section provides a detailed discussion about the incorporation of MOFs and BGs in the same material, emphasizing the benefits that result from their smart combination. In the end, this review shortly explores the promising integration of MOFs and BGs into hydrogels, concluding with a short description of the antibacterial mechanisms of MOFs. For these sections, the keywords “metal-organic framework”, “network”, “zeolite”, “bioglasses”, “hydrogels” and/or “bioactive glasses” were searched for the period 2020–2024 in Scopus, Pubmed, Web of Science and Google Scholar databases. Because of the novelty of this topic resulting in limited published studies so far, in some cases, relevant publications older than 2020 were included in this review paper for completeness. A summary of all the studies detailed in this review is presented in Table 1.

**Table 1:** Summary of the literature mentioned in this review.

	Title	Journal	Year	Technique	BG	MOF type	Main conclusions	Ref.
Comparison of MOFs with BGs	Polydopamine-armored zeolitic imidazolate framework-8-incorporated zwitterionic hydrogel with multifunctional properties for infected wound healing	International Journal of Biological Macromolecules	2024	freeze-drying	–	ZIF-8	• Controlled and slow degradation of Zn^2+^ • Superior antibacterial activity • Promoted fibroblast proliferation and migration • Promoted angiogenesis • Accelerated normal and diabetic wound healing in vivo, while mitigating bacterial growth	[76]
	Enrofloxacin/florfenicol loaded cyclodextrin metal-organic-framework for drug delivery and controlled release	Drug Delivery	2021	–	–	γ-CD-MOF	• High drug loading capacity • Superior short and long-term antibacterial activity • Excellent biocompatibility in vitro and in vivo	[77]
	Metal-organic framework-based nanomaterials for bone tissue engineering and wound healing	Materials Today Chemistry	2022	–	–	Overview of a wide range of MOF compositions	• High porosity and structural stability • Drug release capabilities • Improved antibacterial activity • Stimulated cell attachment, proliferation, migration and differentiation	[78]
	BMP-6 carrying metal organic framework-embedded in bioresorbable electrospun fibers for enhanced bone regeneration	Materials Science and Engineering C	2021	–	–	ZIF-8	• High encapsulation efficiency of BMP-6 molecules in ZIF-8 • Prolonged and controlled release of BMP-6 up to 30 days • Osteogenic differentiation of pre-osteoblasts • New bone formation in vivo	[17]
	Metal–organic framework glass composites	Chemical Society Reviews	2023	–		Overview of a wide range of MOF compositions	• Wide range of pore sizes • High loading capacities • Chemical functionalization • Tailorable composition • Target cell mechanisms • Possible toxicity	[72]
	Oxide Hemostatic Activity	Journal of the American Chemical Society	2006	–	SiO_2_-CaO with different Si:Ca ratios (from 0.25 to 2.5)	**-**	• Reduction of the released heat • Faster clotting	[79]
Synergistic use of MOFs and BGs	3D printed mesoporous bioactive glass/metal-organic framework scaffolds with antitubercular drug delivery	Microporous Mesoporous Materials	2018	3D printing	80SiO_2_-15CaO-5P_2_O_5_ (mol%)	Fe-MOF	• Sustained drug delivery • Enhanced mechanical properties • pH-responsive drug release abilities • Biocompatible and bioactive	[80]
	Drug-loaded zeolite imidazole framework-8-functionalized bioglass scaffolds with antibacterial activity for bone repair	Ceramics International	2021	3D printing	80SiO_2_-16CaO-4P_2_O_5_ (mol%)	ZIF-8	• pH-responsive drug release and antibacterial properties • Enhanced cell proliferation • Stimulated osteogenic differentiation	[18]
	A new MOF@bioactive glass composite reinforced with silver nanoparticles – a new approach to designing antibacterial biomaterials	Dalton Transactions	2024	–	70SiO_2_-30CaO (wt%)	Cu-MOF Ag@Cu-MOF	• Superior bioactivity • Slow and prolonged ion release • Improved biocompatibility • Antibacterial activity	[81]
	Bioactive glass doped with zinc-based metal–organic frameworks (Zn/MOF) nanoparticles for antibiotic delivery applications	Applied Physics A	2024	quick–alkali modified sol–gel method	60SiO_2_-30CaO-10P_2_O_5_ (mol%)	Zn-MOF	• Better dispersity and higher specific surface area • Faster hydroxyapatite formation • Sustained drug release • Biocompatible • Antibacterial properties	[82]
	Characterization of zeolite/bioglass nanocomposites for surface coating of stainless steel material for bone implantation	Journal of Sol-Gel Science and Technology	2022	electrophoretic deposition	63SiO_2_-28CaO-9P_2_O_5_ (mol%)	Commercial zeolite	• Enhanced bioactivity • Improved biocorrosion resistance • Reduced release of metal ions from metal substrate • Promoted osseointegration	[83]
	Fabrication of bioactive glass/chitosan/zeolite bio-nanocomposite: Influence of synthetic route on structural and mechanical properties	Materials Chemistry and Physics	2022	ultrasonic, microwave and liquid phase	45SiO_2_-24.5Na_2_O-24.5CaO-6P_2_O_5_ (wt%)	Commercial zeolite	• Enhanced bioactivity • Improved biocorrosion resistance • Reduced release of metal ions from metal substrate • Promoted osseointegration	[84]
	Enhanced osteogenic activity and bone repair ability of PLGA/MBG scaffolds doped with ZIF-8 nanoparticles loaded with BMP-2	International Journal of Nanomedicine	2023	3D printing	60SiO_2_-36CaO-4P_2_O_5_ (mol%)	ZIF-8	• Slow and sustained growth factor release • Enhanced osteoblast differentiation and proliferation • Promoted new bone formation in vivo	[85]
	Versatile Bioactive Glass/Zeolitic Imidazolate Framework-8-Based Skin Scaffolds toward High-Performance Wound Healing	ACS Applied Materials & Interfaces	2024	microfluidic electrospinning	60SiO_2_-36CaO-4P_2_O_5_ (mol%)	ZIF-8	• Slow and sustained growth factor release • Improved tensile strength • Slow and controlled ion release • Strong antibacterial properties • Promoted growth factor expression and wound regeneration • Higher healing rate	[86]
[]Incorporation of MOFs/BGs into hydrogel	Fabrication of bioactive glass/chitosan/zeolite bio-nanocomposite: Influence of synthetic route on structural and mechanical properties	Materials Chemistry and Physics	2022	including liquid phase, ultrasonic and microwave	45SiO_2_-24.5Na_2_O-24.5CaO-6P_2_O_5_ (wt%)	Commercial zeolite	• Improved fractural and compressive strength • Enhanced bioactivity	[84]
	Composition optimization of Bioactive glass/Chitosan/Zeolite ternary bio- composite	Not officially published	2022	liquid phase method	45SiO_2_-24.5Na_2_O-24.5CaO-6P_2_O_5_ (wt%)	Commercial zeolite	• Improved strength • Enhanced bioactivity	[87]
	Fabrication of bioactive glass 58S/chitosan/zeolite 13X biocomposite using liquid phase method	Journal of Applied Chemistry	2023	liquid phase method	60SiO_2_-36CaO-4P_2_O_5_ (mol%)	Zeolite 13X	• Superior mechanical properties • Faster mineralization	[88]
	3D printing of bone scaffolds based on alginate/gelatin hydrogel ink containing bioactive glass 45S5 and ZIF-8 nanoparticles with sustained drug-release capability	Advanced Engineering Materials	2023	3D printing	45SiO_2_-24.5Na_2_O-24.5CaO-6P_2_O_5_ (wt%)	ZIF-8	• Controlled degradation and drug release • Faster mineralization • Superior cell viability	[89]
	Construction of oxidized icariin functionalized collagen composite with promoting osteogenesis, vascularization and anti-inflammatory properties potential for bone repair application	European Polymer Journal	2024	freeze-drying, crosslinking and freeze-drying	80SiO_2_-15CaO-5P_2_O_5_ (mol%)	Mg-MOF-74	• Enhanced mechanical strength • Delayed degradation • Osteoconductivity and osteogenic differentiation • Stimulated endothelial cell migration • Anti-inflammatory activity	[90]

## 2 Comparison of MOFs with BGs

**Fig. 3:**
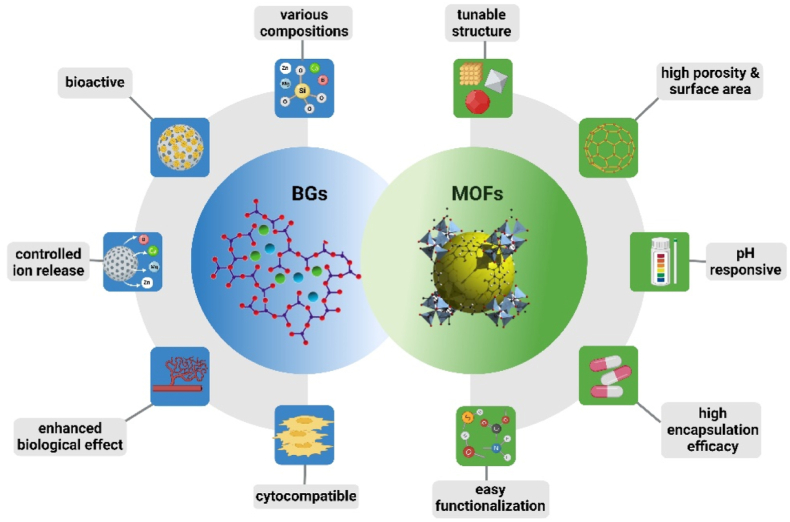
Schematic representation of the main characteristics of BGs and MOFs. Created with BioRender.com.

To gain a better overview about the properties and applications of MOFs/BGs composite materials, it is important to consider first a comparison between these two classes of materials (Fig. 3). Each material has individual features and functionalities, which make them suitable for different uses in the medical field. Their specific properties and characteristics determine how they can be further combined to specifically address different medical challenges. Thus, this section aims to highlight the similarities and differences between these two classes of materials in the biomedical context.

Similar to BGs, MOFs are promising fillers for scaffolds designed for tissue engineering, particularly in stimulating bone regeneration and accelerating wound healing [78]. The high specific surface area, combined with the biocompatibility and tunability of MOFs, play a pivotal role in improving osteoconductivity, osteoinductivity, and wound healing capabilities [78]. Additionally, the high porosity with tunable pore size distribution, high loading capacity, chemical versatility, and high cell-targeting affinity make MOFs (and MOF glasses) ideal candidates for biosensors or imaging contrast agents [72].

When compared to BGs, MOFs have in general higher porosity and smaller pore size, which ensures a high drug encapsulation capacity along with controlled release capabilities. More precisely, MOFs have pore dimensions below 2 nm, whereas for porous BGs the pore sizes are usually exceeding 2 nm. The suitability of MOFs as drug delivery systems is thought to come from the interconnected backbone structures formed by the fusion of metal ions or clusters with organic ligands [77]. Following this assumption, Wei et al. [77] developed γ-CD-MOFs as carriers for florfenicol and enrofloxacin to treat bacterial infections. After synthesizing the γ-CD-MOFs through ultrasonic techniques, the antibiotics were loaded into the pores of the MOFs via physical loading, leading to a controlled and sustained drug release, both in vitro and in vivo, over a relatively long period of time. In comparison to simple antibiotics, the drug-loaded γ-CD-MOF exhibited significantly enhancement of the antibacterial properties. Additionally, the loaded MOFs showed prolonged antibacterial activity, being superior to the use of antibiotics alone. At the same time, the MOFs showed biocompatibility and non-toxicity towards mammalian cells and tissues. Wei et al. [77] also compared these MOFs with MBG particles in the context of drug delivery applications. They referenced the work of Anand et al. [91], who developed ceftriaxone and sulbactam sodium-loaded MBGs with strong antibacterial properties. Similar antibacterial effects were reported by Atkinson et al. [92] for Zn-doped MBG particles, while Pourshahrestani et al. [93] proved that gallium-doped MBGs had both antibacterial and hemostatic properties. Together, these studies are good examples that demonstrate the high potential of using both MBGs and MOFs for controlled drug delivery and ion release [77][].

**Fig. 4:**
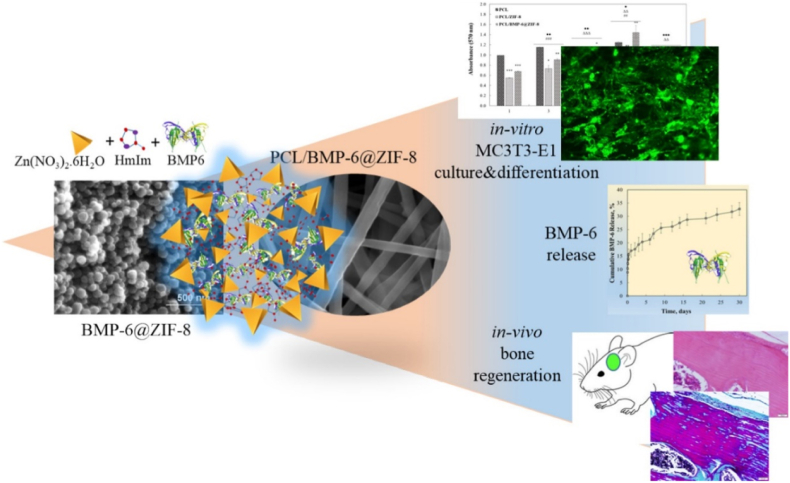
Graphical representation showing the main finding of the study conducted by Toprak et al. [17] in which BMP-6@ZIF-8 MOFs were combined with PCL fibers for bone regeneration. Reproduced with permission from Elsevier [17].

Extending the application of MOFs and MBGs beyond antibiotic drug delivery, recent research has focused on their potential in bone regeneration. For instance, Toprak et al. [17] incorporated bone morphogenetic protein (BMP-6) loaded ZIF-8 (BMP-6@ZIF-8) into poly (ε-caprolactone) electrospun fibers (PCL/BMP-6@ZIF-8) for BTE (Fig. 4). The use of ZIF-8 as nanocarrier for BMP-6 made possible a high encapsulation efficacy of over 98 %, coupled with the capability of controlled release behaviour. In the first 24 h, BMP-6 exhibited a burst release of 12 %, followed by a sustained and controlled release of up to 35 % over the next 30 days. This two-step release profile might arise from the loss of the structural integrity of ZIF-8 in aqueous environments, as well as to the presence of PCL which controls the diffusion of water into the system. The release results were further correlated with in vitro cell studies, showing that the controlled release of BMP-6, influenced by both ZIF-8 and PCL, led to high levels of osteogenic differentiation of MC3T3-E1 pre-osteoblasts. Similar results in terms of cell differentiation were reported by Westhauser et al. [94], who worked on 45S5 BG particles (45 % SiO_2_, 24.5 % Na_2_O, 24.5 % CaO and 6 % P_2_O_5_ (wt %)) [94], observation which highlights the possible similarities between MOFs and BGs in mediating cell behaviour. However, it should be noted that the osteogenic differentiation observed for 45S5 BG was achieved without additional growth factors (most probably as a result of the dissolution products released by the BGs), while the presence of BMP-6 in MOF is likely responsible for the osteogenic differentiation. After demonstrating the beneficial effects of PCL/BMP-6@ZIF-8 in vitro, the researchers performed in vivo tests in critical-sized cranial defect models. In correlation with the previous in vitro results, the PCL/BMP-6@ZIF-8 fibers accelerated the mineralization process involved in the formation of new bone, which was attributed to the presence and controlled release of BMP-6.

Besides bone regeneration, both MOFs and BGs hold significant interest in wound healing applications due to their tailorable functionalities and impressive resistance against phagocytosis and biological degradation [95]. Additionally, the polar surfaces of MOFs and BGs made them suitable for controlling hemorrhages by directly activating specific cellular signalling pathways in the blood coagulation cascade [79]. Even though zeolite-based hemostatic agents (HA) are employed as first-aid equipment for hemorrhages on the battlefield, concerns due to excessive generation of heat upon hydration have been reported [79]. In contrast, BGs-based HA have shown a reduction in the released heat, while acting as rapid agents for promoting clotting. Moreover, by controlling the particle morphology and size, as well as the Si/Ca ratio in BGs, faster coagulation rates can be achieved [79].

Given the antihemorrhagic properties of MOFs, ZIF-8 nanoparticles have been explored as novel fillers in zwitterionic hydrogel to tackle infected wounds [76]. Besides assuring a moist environment needed for wound healing, the poly(acrylamide-co-sulfobetaine methacrylate) (PAS) hydrogel acts as a controller of the release of Zn^2+^ ions from the ZIF-8 structure. To achieve even a longer release of Zn^2+^ and superior adhesive properties at the infected wound site, ZIF-8 particles were coated with a layer of PDA (ZIF-8@PDA). After demonstrating that the PDA coating did not change significantly the physicochemical and morphological properties of the particles, their influence on different physicochemical and biological properties of the PAS hydrogels was assessed. As expected, the presence of the ZIF-8@PDA nanoparticles did not alter the morphology of the hydrogels, all of them exhibiting an interconnected porous macrostructure with ZIF-8@PDA nanoparticles preferentially distributed on the walls of the pores. However, the addition of ZIF-8@PDA significantly increased the tensile strength and elongation, most probably as a result of the electrostatic interactions formed between the PAS hydrogel and the surface of the ZIF-8@PDA nanoparticles. Additionally, when the adhesiveness of these hydrogels was assessed on different substrates (glass, plastic, skin, etc), it was proven that the ZIF-8@PDA containing PSA hydrogel had the highest adhesive strength. This significant improvement was attributed to the presence of the catechol groups in PDA, which could form strong chemical and physical bonds with the substrates. The same functional groups present in the PDA structure conferred antioxidant properties by reacting with the reactive oxygen species (ROS). Despite the beneficial effects of the PDA coating, this coating might have a negative impact on the overall performance of the composite because the PDA layer could act as a barrier for the ion release. However, when antibacterial tests were performed on *E. coli* and *S. aureus,* it was demonstrated that both ZIF-8 PSA and PDA@ZIF-8 PSA had strong antibacterial properties, suggesting that the coating did not impede the release of the Zn^2+^ ions. Moreover, ZIF-8@PDA hydrogels promoted cell proliferation, along with accelerating cell migration and increasing cell density. ZIF-8@PDA MOFs also enhanced angiogenic potential, as demonstrated by the results of tube formation assays. Additionally, ZIF-8@PDA MOFs exhibited exceptional anti-inflammatory properties. When in vivo tests on infected wounds were performed, the ZIF-8@PDA containing hydrogels showed reduced pus formation and bacterial infection and, simultaneously, it accelerated the wound healing process and neovascularization. These results highlight the potential of ZIF-8-containing hydrogels for efficiently treating wound infections.

Despite their advantages, MOFs face several challenges when translated to specific clinical applications, such as implants, fillers of biopolymers or wound dressings [78]. Efforts should be made in optimizing the degradability and stability of these systems according to the physiological conditions of the particular application site and to understand how to target specific cellular behaviour by modifying the composition of MOFs [78]. These issues could be addressed by employing coordination chemistry, which allows precise control over MOFs properties through the use of bioactive linkers [78]. On the other hand, combining MOFs with BGs can improve MOF processability, while assuring superior bioactivity. BGs, in general, offer precise control of ion releasing capability (BG dissolution products) and the possibility to modify the basic chemical composition in the silicate, phosphate or borate systems. In this way, MOF/BG combinations represent composites with promising (multi)functionalities, as discussed in the next section.

## 3 Synergistic combination of MOFs and BGs

As mentioned above, MOFs are widely known for their high surface area, narrow pore size distribution and excellent drug loading capability. Nevertheless, the biological activity and biocompatibility are not always satisfactory [[3], [4], [7], [8]]. In contrast, BGs are recognized for their high bioactivity, characterized by their surface bioreactivity, which further promotes cell adhesion and tissue regeneration [[48], [50], [61]]. Combining MOFs and BGs opens the possibility of taking advantage of the positive attributes of both materials, leading, in the end, to advanced biomaterials with superior properties. This section presents recent work from the literature where the successful integration of MOFs and BGs within the same material was reported, with a focus on their synergistic effect for biomedical applications.

### 3.1 Applications in bone regeneration

**Fig. 5:**
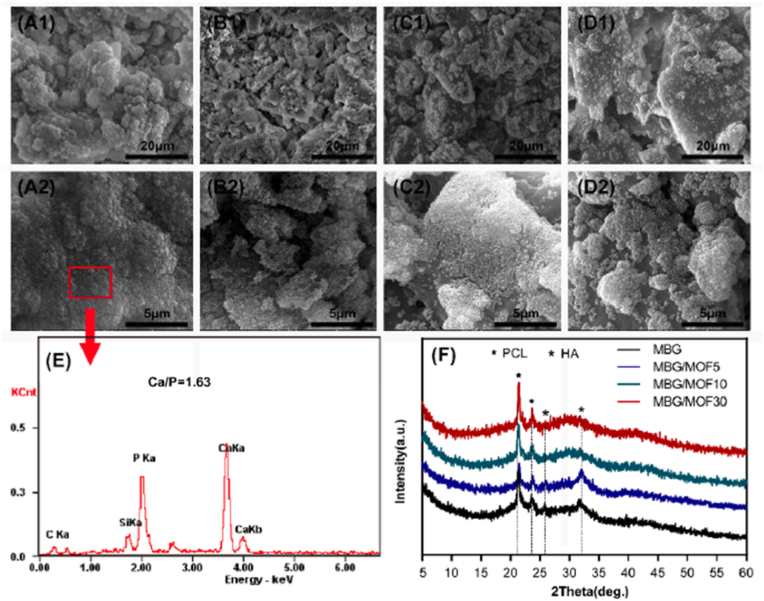
(A1)-(D2) SEM images of MBG scaffolds with increasing MOF content; (E) EDS analysis of MOF/MBG scaffolds; (F) X-ray diffraction patterns of both MBG and MOF/MBG scaffolds after 3 days of immersion in SBF solution, according to Pei et al. [80]. Reproduced with permission from Elsevier[80].

If the pH-responsive properties of MOFs made them ideal for controlled drug delivery, the intrinsic ability of BGs to stimulate bone formation makes them excellent candidates for bone repair and regeneration. The integration of MOFs and BGs therefore enables the development of composite scaffolds that not only possess controlled and sustained drug release, but also have appropriate mechanical properties and tailorable degradation rates. To take advantage of both materials, Pei et al. [80] developed 3D printed scaffolds containing isoniazid-loaded MBGs and MOFs for the treatment of osteoarticular tuberculosis. After synthesizing the MBGs by a modified sol-gel method, the ordered mesoporous structure was confirmed by transmission electron microscopy. These results were further correlated by N_2_ adsorption-desorption analysis, revealing a high surface area of 395 m^2^/g and a pore volume of 0.40 cm^3^/g, which could suggest enhanced bioactivity and high drug loading capability. In the next step, inorganic pastes with increasing ratios of MBGs and MOFs were prepared and then used to fabricate scaffolds via 3D printing. The presence of the MOFs did not change the macrostructure of the scaffolds, all of them having a similar macroporosity with pores of approximately 400 μm. Furthermore, in vitro bioactivity studies in simulated body fluid (SBF) proved that all of them exhibited bioactive properties despite adding different amounts of MOFs (Fig. 5). However, increasing MOF content led to a decrease in the pH of the SBF, most probably due to the release of acidic degradation byproducts from MOFs. On the other hand, higher MOF concentrations increased the compressive strength and accelerated the degradation rate of the scaffolds, which could further change the drug release rate. To investigate this hypothesis, isoniazid (INH) was loaded in the mesopores and on the surface of the MBG powder. Despite the variation in MOF content, INH was released in a similar way from all scaffolds, showing a two-step release profile. Nevertheless, significant differences were noticed in the initial burst release phase, with higher MOF content leading to an increase in the INH release. This observation can be correlated with the results of the degradation studies, where faster degradation was recorded for higher MOF concentrations, which might have generated additional diffusion pathways that could accelerate the INH release. In the second release stage, all scaffolds exhibited a sustained INH release, probably due to the confinement provided by the mesoporous channels of the MBG, demonstrating once again the synergistic effect of combining MBGs with MOFs. Moreover, the well spread morphology of human bone marrow-derived mesenchymal stem cells (hBMSCs) on the surface of the scaffolds proved the biocompatibility of all samples. Overall, this study demonstrated the potential use of MOF/MBG composite scaffolds for advanced drug delivery and bone regeneration applications, opening new research pathways for novel treatments of osteoarticular conditions.

**Fig. 6:**
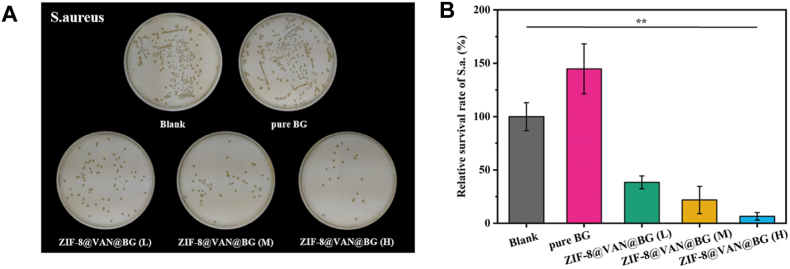
Assessment of the in vitro antibacterial effect of BG and ZIF-8@VAN@BG scaffolds. (A) Images depicting *S. aureus* colonies after treatment with various scaffolds; (B) survival rates of *S. aureus* following treatment with different scaffolds, according to Han et al. [18]. Reproduced with permission from Elsevier [18].

A similar approach that takes advantage of the synergistic effect of combining MOFs and BGs was proposed by Han et al. [18] for treating bone infections. In this regard, the researchers fabricated BG scaffolds via 3D printing, which, after sintering, were used for in situ deposition of ZIF-8. The use of ZIF-8 could assure high chemical stability in neutral environments and controlled degradation in acidic environments [96]. In this way, ZIF-8 becomes a promising pH-responsive nanocarrier for the delivery of antibiotics. During the synthesis of the ZIF-8 nanoparticles, different concentrations of vancomycin were incorporated to confer antibacterial properties to the scaffolds, specifically targeting the growth inhibition of *S. aureus* and the prevention of biofilm formation. After producing the vancomycin-loaded ZIF-8 nanoparticles (ZIF-8@VAN), they were deposited on the BG scaffold's surface (ZIF-8@VAN@BG) and characterized in terms of physicochemical and biological properties. X-ray diffraction (XRD) confirmed the successful incorporation of ZIF-8 and ZIF-8@VAN on the BG scaffolds by the identification of the characteristic crystalline peaks of ZIF-8, in contrast with simple BG samples where the expected amorphous structure was confirmed. The XRD results align well with the FTIR spectra, where the imidazole ester group vibrations proved the presence of ZIF-8 within the materials. Although both BG and ZIF-8@VAN@BG scaffolds maintained an open porosity of approximately 75 %, the presence of ZIF-8@VAN nanoparticles led to significant changes of the surface morphology. While on the surface of the BG scaffolds spherical particles with gaps between them were identified, for the ZIF-8@VAN@BG scaffolds these empty spaces were filled with ZIF-8@VAN nanoparticles. The surface coverage of ZIF-8@VAN increased proportionally with their concentration. Despite different microstructural morphologies, both BG and ZIF-8@VAN@BG scaffolds had similar compressive strengths, indicating that the structural integrity of the scaffolds was not affected by the deposition of the ZIF-8@VAN nanoparticles. To confirm the acid-responsive behaviour of ZIF-8@VAN@BG scaffolds, the samples were immersed in phosphate buffered saline solutions (PBS) at pH 5.4 and 7.4. Even though under both conditions vancomycin had a similar two-step release profile, the amount of drug released was different. More precisely, in the acidic environment, the relative vancomycin release was over 70 %, whereas at neutral pH, it reached only 50 %. This behaviour highlights a pH-controlled release mechanism, as a consequence of the enhanced degradation of ZIF-8 in acidic conditions. The degradation study, performed in tris(hydroxymethyl)aminomethane (TRIS), revealed that the process was slowed down when ZIF-8@VAN particles were incorporated into the BG scaffolds. The authors attributed this delay to the presence of ZIF-8 which is stable in physiological conditions, hypothesis further supported by the low variation of the pH of the TRIS solution. Results showed that ions were slowly released from the ZIF-8@VAN@BG scaffolds, which also exhibited slow degradation, proving the potential of using these systems for achieving enhanced structural and fine dependent functional integrity. When cell compatibility tests were performed on rat bone marrow mesenchymal stem cells (rBMSCs), a significant increase of the rBMSCs cell proliferation was observed for ZIF-8@VAN@BG scaffolds compared to BG scaffolds. Furthermore, the relative cell viability of rBMSCs increased with increasing ZIF-8@VAN concentration. Considering the cellular mechanisms behind this process, the researchers proved that the presence of ZIF-8@VAN promoted the differentiation of rBMSCs by upregulating osteogenic gene expression. On the other hand, the ZIF-8@VAN@BG scaffolds exhibited improved antibacterial properties against *S. aureus* (Fig. 6). This effect might come as a result of the controlled release of vancomycin, showing another advantage of adding ZIF-8@VAN nanoparticles to BG scaffolds. Thus, these pH-responsive ZIF-8@VAN@BG scaffolds hold great promise for infected bone repair applications, especially because of their high antibacterial activity and biocompatibility. The simultaneous integration of BGs and MOFs can assure a sustained release of antibiotics, without affecting the scaffold's structural integrity, being possible to decouple drug release kinetics from degradation behaviour for supporting more efficiently bone regeneration. Hence, this study is another example of the potential of these innovative MOF/BG composites for developing advanced scaffolds.

If in the previously presented studies BG scaffolds served as a support for drug-loaded MOF particles, BGs and MOFs can be also integrated into the same matrix material. For instance, Ahmed et al. [82] reported the successful synthesis and characterization of Zn-doped MOFs/BGs (Zn-MOFs/BGs) nanocomposite nanoparticles, designed for the targeted delivery of gentamicin to treat bone infections. Obtained by a quick-alkali-modified sol-gel method, the BG was synthesized by mixing the precursors with the solvent and catalyst in a solution in which ZIF-8 nanoparticles had been previously dispersed. Both BG (composition: 60 % SiO_2_-10 % P_2_O_5_-30 % CaO (wt%)) and Zn-MOFs/BGs (60 % SiO_2_-10 % P_2_O_5_-29.2 % CaO-0.8 % Zn-MOFs (mol%)) nanoparticles had a compact morphology with diameters below 100 nm. Among them, the Zn-MOFs/BGs nanoparticles showed a slight increase in particle size and a higher degree of dispersity. Surprisingly, although crystalline Zn-MOFs were incorporated into amorphous BGs, the XRD analysis showed the characteristic pattern of an amorphous material, most probably because of the low amount of Zn-MOFs. However, the specific surface area and the pore diameter of the nanoparticles increased after adding Zn-MOFs, further highlighting the suitability of Zn-MOFs/BG as novel nanocarriers for treating bone infection. Subsequent in vitro experiment in SBF demonstrated faster mineralization for Zn-MOFs/BGs. Given the promising morphological, structural and textural properties of these Zn-MOFs/BGs nanocomposite particles, the second part of the study explored their use as drug delivery systems for the controlled release of gentamicin. Even though Zn-MOFs/BGs nanoparticles have a higher specific surface area compared to BGs, they did not show a significantly higher loading capacity of gentamicin. However, an important difference was observed in terms of their drug release profiles. When Zn-MOFs were incorporated into BGs, the initial amount of drug released was significantly reduced (from 31 % to 16 %). This change might come from the porous nature of MOFs which could prevent and delay the diffusion of the drug out of the material. Similarly, after 28 days of evaluation, BGs/Zn MOFs showed a reduction in the amount of drug released compared to BGs. Overall, both Zn-MOFs/BGs and BGs had a similar two-stage release profile, described by the Higuchi model, confirming the sustained release ensured by the composite Zn-MOFs/BGs particles. In the next step, MG-63 osteoblast-like cells were used to assess the cytotoxicity of the MOFs/BGs particles at two different concentrations, 100 μg/ml and 300 μg/ml. The quantitative data revealed that the materials were not cytotoxic, while in the microscopy images no important changes in the morphology of the MG-63 cells were observed. At the same time, both BGs and Zn-MOFs/BGs were found to have antimicrobial properties against *E. coli, S. aureus* and *Candida albicans*. Nevertheless, Zn-MOFs/BGs exhibited significantly higher antibacterial abilities than BGs. This enhanced antibacterial property is likely the result of the release of Zn^2+^ ions, which are recognized for their strong antibacterial properties. In conclusion, the addition of Zn-MOFs to BGs improved the mineralization and antibacterial activity of these materials, while achieving a controlled drug release along with a high cell viability. These findings prove the potential of Zn-MOFs/BGs nanocomposites as therapeutic materials for bone infection treatment, having complementary functional properties which do not interfere with their biocompatibility.

Another approach of combining BGs and MOFs for treating bone infections was proposed by Fandzloch et al. [81]. The authors coated sol-gel derived 70 % SiO_2_-30 % CaO (mol%) BGs pellets with a thin layer of Cu-MOFs onto which Ag nanoparticles were directly synthesized by reducing AgNO_3_ precursor. In the first step, the amorphous nature of the compact BG nanoparticles, with spherical shapes and diameters around 70 nm, was confirmed. Simultaneously, NH_4_[Cu_3_(μ_3_-OH)(μ_3_-4-carboxypyrazolato)_3_] MOFs were used as a support for growing Ag nanoparticles (Ag@Cu-MOFs). Even though XRD diffraction patterns did not reveal crystalline Ag, probably due to the low nanoparticle content, transmission electron microscopy images and elemental mapping confirmed the uniform distribution of 10 nm Ag nanoparticles on the surface of the Cu-MOFs. Despite maintaining the micro- and mesoporosity of the Cu-MOFs, the specific surface area and pore volume were reduced to half for Ag@Cu-MOFs. After the successful characterization of the BG and Ag@Cu-MOFs nanoparticles, BG nanoparticles were pressed into discs and spin coated with Ag@Cu-MOFs. The obtained BG@Ag@Cu-MOFs composites showed delayed mineralization compared to pure BGs, which might be caused by the presence of the Ag@Cu-MOFs coating that acts as a barrier, hindering the ion release. However, in the XRD patterns, calcium phosphate peaks were identified after three days, while following seven days of incubation a needle-like precipitate was identified on the surface of the pellets. As expected, the Ag@Cu-MOFs nanoparticles had a superior antimicrobial efficacy compared to Cu-MOFs, with a significant stronger effect against Gram-negative bacteria than Gram-positive strains and yeasts. Nevertheless, for the BG@Ag@Cu-MOF nanocomposites, a high antibacterial effect against all strains and yeasts was recorded, proving the synergistic effect of the simultaneous release of different ions. Indirect cell studies on human dermal fibroblasts showed no changes in cell viability for BG@Ag@Cu-MOF, while cell migration was stimulated without altering the cell phenotype.

**Fig. 7:**
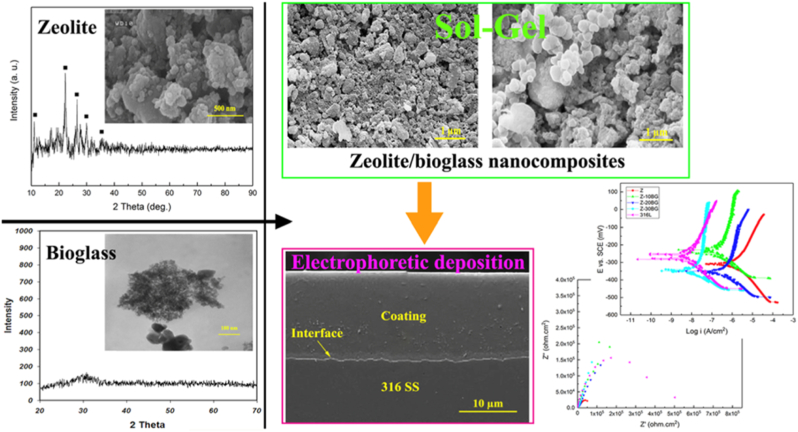
Summary of results of the study of Zhao et al. [83], showing the successful synthesis of zeolite and BG materials along with the improvement of the electrochemical properties of BG/zeolite coated 316 substrates. Reproduced with permission from Springer Nature [83].

To combine the benefits of MOFs and BGs for orthopaedic applications, Zhao et al. [83] focused on improving the surface properties of 316L stainless steel implants by depositing on their surface a thin layer of zeolite/BG (Fig. 7). BG with the nominal composition 9 % P_2_O_5_–28 % CaO–63 % SiO_2_ (wt%) was used. After magnetically stirring different ratios of zeolite and BG for 10 min, the resulting zeolite/BGs suspensions were deposited on the metal substrates by electrophoretic deposition (EPD). In order to find the coating with the optimum characteristics, different voltages and heat treatments were evaluated. Thus, high voltage led to cracks, uncontrolled porosity, and particle agglomeration, while appropriate heat treatment ensured a strong adhesion between the substrate and the coating, without having a negative influence on the integrity of the deposited layer. Ultimately, when applying the optimum voltage and temperature, a crack-free and uniform zeolite/BG coating was successfully deposited on 316L stainless steel substrates using EPD. Increasing the content of BG nanoparticles not only enhanced the quality of the coating, but also improved its uniformity. When tested for bioactivity in SBF for 21 days, the zeolite/BG-coated substrates showed superior bioactivity compared to the uncoated sample, as demonstrated by the thicker HAp layer formed. Additionally, with higher BG content the mineralization process was accelerated, highlighting in this way the advantage of combining BG and zeolites for achieving better biological properties. To evaluate the protective qualities and the resistance against biocorrosion of the zeolite/BG coatings, Tafel polarization and electrochemical impedance spectroscopy tests were employed. The presence of the coating led to a superior corrosion resistance in biological environment, resistance which increased for higher concentrations of BG particles. In this way, the release of toxic metal ions coming from the substrate can be hindered by the presence of the coating, while the ions released from the same coating may have therapeutic effects on promoting osteointegration. Thus, it can be concluded that combining zeolite/BG nanocomposites with EPD offers a promising approach to develop multifunctional coating to enhance the properties of bone implants. This method combines the corrosion protective and bioactive properties arising from the zeolite/BG coating with the simplicity and cost-effectiveness of the EPD process.

In the quest to find new solutions for BTE, the simultaneous integration of MOFs and BGs in the same polymer shows promising potential. Based on the success of previous studies using MOF/BG composites for BTE, Ma et al. [85] fabricated novel porous poly (lactide-co-glycolide) (PLGA)/MBG composite scaffolds containing bone morphogenetic protein-2 (BMP-2) loaded ZIF-8 particles by 3D printing technology. While the BMP-2 was incorporated into the MOF nanocarrier during the synthesis of the ZIF-8, MBGs were prepared separately by a modified sol-gel method. Subsequently, the MBG powders and BMP-2 loaded ZIF-8 were added to the PLGA solution, and the obtained mixtures were processed by 3D printing. The results revealed that ZIF-8, known for its high porosity and tunability, served as an effective carrier for BMP-2, demonstrating sustained release of BMP-2 without an initial bursting. Approximately 40 % of BMP-2 was released within the first 72 h, followed by a sustained release for up to 174 h. It was assumed that a third release phase might occur after the complete degradation of ZIF-8 and PLGA/MBG scaffolds, corresponding to the liberation of the remaining BMP-2. Besides controlling the release of BMP-2, the addition of the ZIF-8 particles significantly increased the compressive strength of the scaffolds. This improvement was attributed to the reinforcement effect of the ZIF-8 particles which fill the empty spaces present in the microstructure of the initial scaffold. Furthermore, the spreading and proliferation of MC3T3-E1 cells were higher for the PLGA/MBG/ZIF-8/BMP-2 (PMZB) scaffold, which was thought to be a consequence of the increased hydrophilicity, MBG degradation products, ZIF-8 incorporation and BMP-2 loading. Furthermore, the evaluation of the osteogenic differentiation proved that the PMZB scaffold could increase the ALP expression together with upregulating specific osteogenic genes, which leads, in the end, to increased formation of calcium nodules. These outcomes might come from both ZIF-8 particles and MBGs. In the last step, in vivo assessment using rat calvarial bone defect models confirmed the ability of the PMZB scaffold to repair and improve the formation of new bone, most probably as a result of the release of BMP-2. Thus, the PMZB scaffold, with compressive strength comparable to human cancellous bone, proves to be a superior osteoconductive and osteoinductive bone substitute. These PMZB scaffolds have the potential to reduce the BMP-2 dosage and cost, while avoiding heterotopic ossification. Therefore, the study underscores the potential of the PMZB scaffold as an effective and cost-efficient solution for BTE, combining sustained growth factor release with appropriate mechanical properties.

### 3.2 Applications in soft tissue regeneration

Recent advances in BG/MOF materials enabled their transition from bone tissue regeneration to soft tissue engineering applications, owing to the release of therapeutic ions from their structure. For instance, Hou et al. [86] investigated the incorporation of BG and ZIF-8 particles into PCL/poly(vinyl alcohol) (PVA) fibers obtained by microfluid electrospinning to fabricate multifunctional scaffolds for wound healing. The BG particles, synthetized through a sol-gel process, had a spherical morphology and negative surface charge, whereas ZIF-8 exhibited polyhedral shape with positive zeta potential. The difference in surface charge between the two components could favour the formation of electrostatic interactions between them. When added to the PCL/PVA fibers, the BG/ZIF-8 particles did not change the interconnected fiber network, but increased the surface roughness and enhanced the tensile strength, concurrently with a reduction of elongation at break. The simultaneous ion release from BG particles and ZIF-8, resulting in high values of the pH, has been shown to confer the composite fibers superior antibacterial properties against *E. coli* and *S. aureus*. Despite showing cytotoxicity on L929 fibroblasts, most probably due to high ion concentrations, the BG/ZIF-8 fibers significantly accelerated the wound healing process and increased the thickness of the granulation tissue, leading to complete scab formation and necrotic tissue reduction, together with accelerated angiogenesis. The enhancement of the regeneration process could be attributed to the therapeutic effect of the released ions, which can further stimulate specific cellular pathways. Even though the research on BG/MOFs for soft tissue engineering is not yet fully explored, this study highlights the high potential of exploiting the multifunctional benefits of BG and MOFs in this sense.

All the studies presented in this section confirm the impressive advancements that can be achieved by combining MOFs and BGs for biomedical purposes. MOF/BG composites have emerged as innovative and efficient solutions for high-performance therapies, by taking advantage of the high surface area and flexibility of MOFs together with the bioactivity and biocompatibility of BGs. This synergistic enhancement has enabled not only the development of personalized drug delivery systems, with increased mechanical strength, but also it stimulated the proliferation and differentiation of different types of cells. Considering the success of these composites reported so far, future research directions should focus on incorporating MOFs and BGs in other materials, such as hydrogels, expanding in this way their potential use in regenerative medicine and beyond. Given the increasing popularity of hydrogels in tissue engineering applications due to their structural and functional similarities to native tissue, in the next section, we focus on the simultaneous incorporation of MOF/BG systems into hydrogels. In this way, an in-depth analysis of these promising composites is provided, outlining their significant potential for biomedical applications.

## 4 Incorporation of MOFs/BGs in hydrogels

In the past years, hydrogels have become important biomaterials for tissue engineering applications, owing to their structural similarities to native tissue [36]. More precisely, they possess high biocompatibility and versatility, which enables the development of materials with tunable physicochemical properties, while supporting cell growth by closely mimicking the structure of the extracellular matrix [36]. Additionally, hydrogels can be combined with different inorganic materials to enhance their properties. In this sense, the incorporation of MOFs and BGs into hydrogels holds significant promise for developing systems with targeted and sustained drug release, while stimulating normal tissue regeneration processes [97]. Although this review is not primarily focused on this topic, this section aims to provide a brief overview of important research studies that integrate MOFs/BGs into hydrogels. Therefore, the following studies will be mentioned only in a short, summarized version, offering the reader a wide perspective on the further applications of MOFs/BGs in the biomedical field.

To illustrate the potential of such MOFs/BGs/hydrogels for medical applications, Moghaddam et al. [84] have recently reported the development of ternary composites containing different proportions of BGs, chitosan, and zeolite. The authors investigated the influence of different synthesis methods, namely ultrasonic, liquid phase processing and microwave techniques, on the properties of zeolite/BG/chitosan nanocomposites. In these systems, the zeolite content was maintained constant, while the weight percentages of BG particles and chitosan were varied, further investigating how the different BG and chitosan ratios affect the overall properties of the composites. The results of the mechanical tests revealed that composites containing medium amounts of BG and chitosan, synthesized by liquid phase route, had the highest fracture toughness and compressive strength. However, the composites obtained via liquid phase processing showed superior mechanical properties compared to those obtained by the other methods. The bioactivity of the samples increased with increasing the content of BG, highlighting the importance of the added BG particles in accelerating the mineralization process. Based on these promising results, the same authors further optimized the BG/chitosan/zeolite ternary biocomposite to achieve better mechanical and biological properties (online report [87]). The incorporation of the zeolite and BG within the chitosan matrix was confirmed by XRD and FTIR analysis, while the compressive strength of the samples increased with increasing BG concentration. Another beneficial effect of increasing BG content was observed after performing bioactivity tests in SBF, with the results demonstrating an increase in the formation of a HAp layer on the surface of the scaffolds immersed in SBF after only 3 days. In contrast to BG results, it was shown that the amount of added zeolite did not significantly influence the mineralization capacity of the samples, but it did increase the Young's modulus. Similar improvements of compressive strength and biomineralization capacity were reported by the same group for BG/13X zeolite/high molecular weight chitosan nanocomposites [88]. All these studies showcase the high potential of incorporating BG, zeolites and chitosan in advanced biomaterials, emphasizing their enhanced mechanical properties and improved bioactivity for tissue engineering applications.

**Fig. 8:**
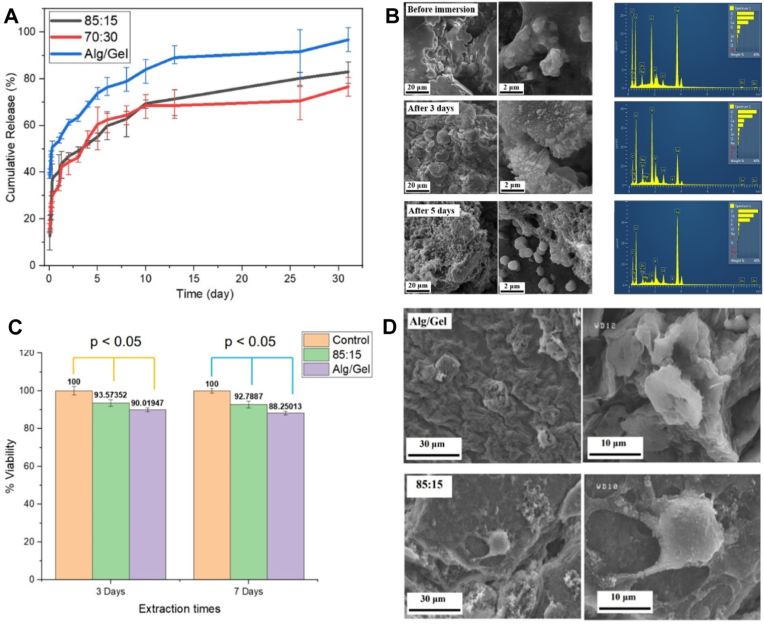
(A) Cumulative Dex release from Alg/Gel scaffolds containing different ratios of MBG/ZIF-8; (B) in vitro SBF test results; (C) MTT cell viability results and (D) SEM images of the cells within the scaffold, according to the study of Anvari Kohestani et al. [89]. Reproduced with permission from John Wiley and Sons [89].

To further extend the properties of BGs/MOFs/hydrogel systems, recent research focused on combining these materials with cutting-edge fabrication techniques. In this regard, Anvari Kohestani et al. [89] developed 3D printed alginate/gelatin (Alg/Gel) scaffolds containing 45S5 MBG and ZIF-8, intended for BTE. Even though MBGs stand out due to their ordered mesoporous structure and high porosity, they face challenges in terms of mechanical strength and 3D printability. To overcome these drawbacks, MBGs were integrated into an Alg/Gel matrix, known for its compatibility and printability. The complexity of this system was further increased by the addition of ZIF-8, to exploit its large surface area and the ability to adjust the drug release, making it suitable as an anti-inflammatory drug carrier. Considering the inflammatory responses post-implantation, dexamethasone (Dex), an anti-inflammatory drug, was loaded in ZIF-8. If the final concentration of MBGs and ZIF-8 with respect to Alg/Gel was kept constant, two different MBG:ZIF-8 mass ratios (85:15 and 70:30) were studied. After preparing the corresponding inks, they were characterized in terms of their rheological properties. The results revealed a similar non-Newtonian fluid behaviour for both 85:15 and 70:30 compositions, with the storage modulus indicating that the scaffolds could maintain their shape upon printing. Even though changing the ratio between MBG and ZIF-8 did not induce rheological changes, the nanocomposite bioinks had lower storage and loss moduli compared to plain Alg/Gel, suggesting that MBG/ZIF-8 could enhance the mechanical properties during printing. The presence of MBG/ZIF-8 did not induce changes in the macrostructure of the 3D printed scaffolds, which exhibited an interconnected porosity with well-aligned struts. On the other hand, modifications of surface topography were observed. More precisely, the presence of MBG/ZIF-8 led to an increase in surface roughness. Regarding the ultimate shear strength (USS), increasing the MBG content with respect to ZIF-8 led to a higher USS, which was attributed to the possible higher inhomogeneity of the 70:30 containing Alg-Gel ink. At the same time, increasing the MBG content reduced the swelling capacity of the scaffolds, but, interestingly, it did not affect the cumulative release of Dex. All scaffolds showed a burst release in the first 10 h and a sustained and constant cumulative release up to 31 days. Compared to plain Alg/Gel, the MBG/ZIF-8 containing scaffolds reached a lower cumulative release at all time points, demonstrating the benefits of loading Dex inside the ZIF-8 to control its release (Fig. 8A). Furthermore, the MBG/ZIF Alg/Gel scaffolds exhibited bioactivity in SBF, as evidenced by the HAp layer formed on their surface (Fig. 8B). The cytotoxicity assay, performed for 3 and 7 days on MG-63 osteoblast-like cells, confirmed the cytocompatibility of the MBG/ZIF-8 containing scaffolds (Fig. 8C). These findings were further supported by SEM images showing cells attached to the scaffolds, all of them possessing clear signs of filopodia formation (Fig. 8D). It can be concluded that by carefully adapting the ratio between MBG and ZIF-8 added to the Alg/Gel 3D printed scaffolds, structures with improved physicochemical, morphological, drug release and cytocompatibility properties can be achieved, showing great potential for BTE.

More recently, Li et al. [90] reported the promising pro-osteogenic, pro-vascularization and anti-inflammatory properties of collagen (Col)/amino functionalized BGs (BG-NH_2_)/MOFs scaffolds. BG particles of composition 80 % SiO_2_-15 % CaO–5% P_2_O_5_ (mol%) were used. BGs were functionalized with amino groups to improve the interfacial bonding with Col, while Mg-MOF-74 was chosen for its reinforcing and angiogenic potential of the released Mg^2+^ ions. To ensure the long-term stability and higher efficacy of Mg-MOF-74, this MOF was coated with a thin layer of mesoporous SiO_2_. Additionally, oxidized icariin (OICA) was incorporated into the scaffold, serving both as a crosslinker between Col and BG-NH_2_ and imparting anti-inflammatory properties. After fabricating and crosslinking the scaffolds in a two-step freeze-drying process, the samples were characterized in terms of crystallinity, morphology, mechanical properties, cytotoxicity, and anti-inflammatory properties. Thus, the XRD patterns and the FTIR spectra confirmed the successful incorporation of BG-NH_2_ and Mg-MOF-74 in the collagen scaffolds, which maintained an interconnected porous network needed for cell adhesion and vascularization. However, adding BG-NH_2_ increased the surface roughness, while the presence of OICA led to a decrease in porosity. Despite showing slight morphological changes, OICA-containing scaffolds had a higher Young's modulus and delayed collagenase degradation, most probably as a result of the crosslinking effect of OICA. When it comes to the bioactivity of the scaffolds, the addition of Mg-MOF-74 to the Col/BG-NH_2_ scaffolds decreased their mineralization capacity. This reduction was attributed to Mg^2+^ ions released from the Mg-MOF-74, which can be adsorbed by negatively charged binding sites in Col, thus, decreasing the number of places available for Ca^2+^ adsorption. The cell studies, performed using BMSCs, showed an enhanced proliferation and a higher ALP activity after adding Mg-MOF-74 to the Col/BG-NH_2_ scaffold, confirming the beneficial therapeutic effect of Mg^2+^. Furthermore, the Col/BG-NH_2_/Mg-MOF-74 exhibited a higher anti-inflammatory activity than Col/BG-NH_2_. Ultimately, the simultaneous incorporation of BG-NH_2_ and Mg-MOF-74 into collagen hydrogel enhanced the structural, osteogenic, and anti-inflammatory properties of the scaffolds, reinforcing its suitability for BTE applications.

## 5 Simultaneous enhancement of antibacterial and biological properties in MOFs/BGs composites

The assessment of toxicity plays a pivotal role in designing novel biomaterials and the importance of understanding the cytotoxicity of MOFs is particularly emphasized in the literature. Nevertheless, discussions on MOFs toxicity are limited, with available information often being focused on the inorganic and organic precursors rather than the MOFs themselves. Fe, Al, and Cr based MOFs, such as MIL-100 and MIL-88, are more likely to be toxic and are more commonly investigated in this regard [[98], [99]]. In vitro studies of these MOFs showed minimal cytotoxic effects, whereas in vivo administration of high doses of Fe-MOFs to rats did not reveal severe toxicity [99]. Moreover, the cytotoxicity of MOFs is further correlated with their composition, but it remains relatively low compared to other commercialized nanosystems [[98], [99]]. The gradual degradation of MOF structures together with the slow release of their components, contribute to reducing the transient concentrations suggesting that by tailoring MOF components, materials with lower cytotoxicity and an improved safety profile can be developed [98].

In this context, Liu et al. [100] explored MOFs as emerging hybrid nanoparticles composed of metal cations and organic linkers with special physicochemical properties. MOFs, renowned for their high surface area, flexible structure, and tunable pore sizes, have also gained significant attention, particularly for their intrinsic antibacterial properties. Recent studies focused on MOFs have highlighted their high antibacterial efficacy, further complementing their physicochemical properties, biocompatibility and biodegradability [[100], [101], [102]]. The ease of functionalization facilitates the fabrication of different types of nanocomposites, enhancing antibacterial applications through synergistic effects. Several antibacterial applications of MOFs have been reported, including imparting long-lasting antibacterial properties to membranes with minimal rates of loading, providing self-cleaning functionality for wound infection management, acting as a biofilm inhibitor in BTE, and serving as efficient antibacterial agents for water treatment [103].

**Fig. 9:**
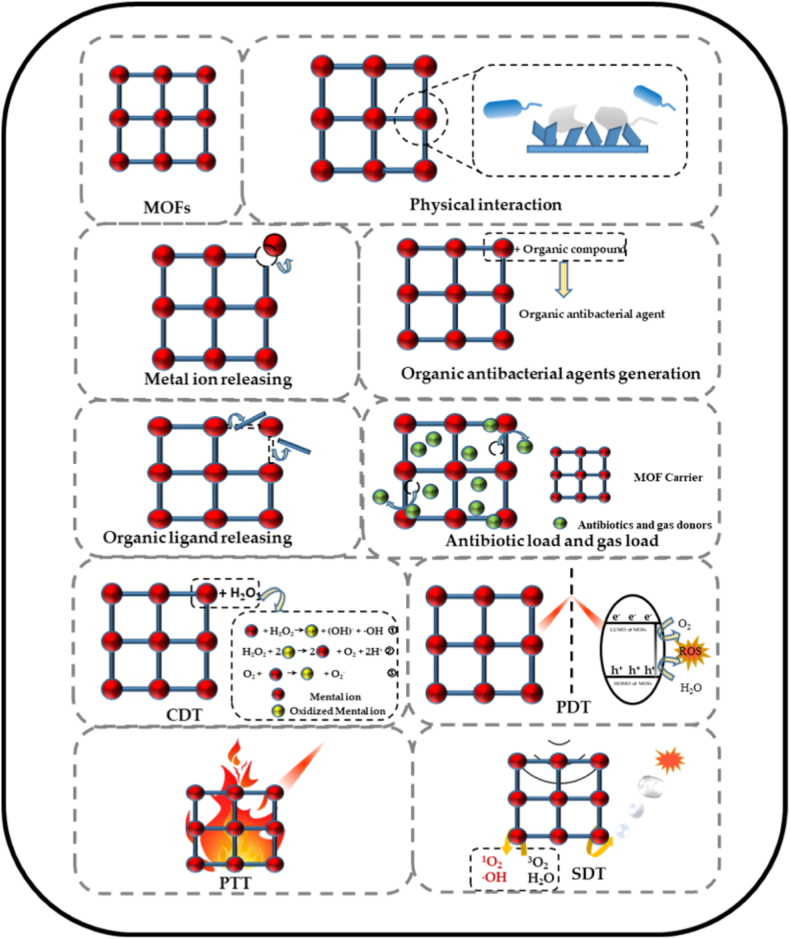
Main antibacterial mechanism of MOFs (CDT - chemical dynamic therapy, PDT - photodynamic therapy, PTT - photothermal therapy, SDT **-** sonodynamic therapy). Reproduced from Zhang et al. [101] under a Creative Commons CC BY License.

MOFs are employed in antibacterial applications through different methods. The first extensively researched technique involves utilizing MOFs as carriers for antibacterial substances, owing to their intrinsic porous structure, which offers a high loading capacity and a sustained release profile [78]. The second approach uses the direct action of MOFs as antibacterial agents, with the antibacterial mechanisms being categorized into physical contact, metal cations and ligands release, photothermal effects, oxidative stress, and synergistic impacts (Fig. 9) [[100], [101]]. However, behind all these mechanisms, their significant antibacterial activity originates from the release of metal cations and organic ligands. For instance, the release of various metal ions such as Ag^+^, Zn^2+^, Cu^2+^ and Co^2+^ cations upon immersion in aqueous environments holds great promise in addressing the challenges related to infection and bacterial biofilm formation, due to their broad-spectrum antimicrobial activity and different mechanisms of action [[104], [105]]. Similarly, the antibacterial properties of BGs, including MBGs, are usually linked to their ability to release the same antibacterial ions (Ag, Zn or Cu) [52]. Even though in the end similar antibacterial effect can be achieved, the antibacterial mechanism of action varies from one cation to another. Among them, the most important are DNA and protein damage, membrane permeabilization, accumulation of antibacterial agents, generation of radicals, disruption of proton motive force, enzyme inactivation, and endocytosis [[98], [100], [101], [104]]. For example, cobalt ions tend to bind to anions such as PO_4_^3−^ present in the phospholipid membrane, inducing pro-oxidative stress and, concurrently, generating reactive oxygen species (ROS) to impair bacteria [100]. On the other hand, the release of organic ligands from MOFs under physiological conditions also contributes to the antibacterial activity, as observed in a Zn-based MOF inhibiting *S. aureus* growth [101]. The antibacterial properties of these ligands result from their binding to Ca and Mg cations within bacterial cells, causing the fragmentation of cellular DNA. Furthermore, the controlled and sustained release of metal cations and ligands contributes to the long-lasting antibacterial durability of materials [[100], [101], [104]]. In this context, further research should explore the synergistic antibacterial effects between the simultaneous release of the metal ions and the ligands present within MOF structure [103]. By carefully controlling the degradation behaviour of MOFs, an enhancement of their antibacterial efficacy and broadening of their potential applications can be achieved, while it can be further enhanced by smart combination with ion release BG nanoparticles [103]. Indeed, constructing MOF-based composites represents a third approach for employing MOF in antibacterial applications, emerging as a promising strategy to synergistically enhance the intrinsic antibacterial effects compared to individual components. The combination of MOFs with different materials such as metals, oxidation products, carbon-based materials, BGs and other MOFs has been shown to significantly improve their antibacterial effect compared to standalone MOFs [101]. For instance, 2D MOF nanosheets decorated with Ag nanoparticles demonstrated promising antibacterial properties against *E. coli* and *S. aureus*, attributed to the synergistic release of the MOFs components and the photodynamic release of Ag^+^ [106]. Similar enhanced chemodynamic antibacterial effects were reported for CuS nanoparticles coupled on the surface of Co-ferrocene-MOF nanosheets on the same bacterial strains [107]. Additionally, MOFs have been also used in polymer research, where they are used together with polymers to create nanocomposites which act as an exceptional platform for multifunctional antibacterial materials. These systems are capable of controlled polymerization, developing novel hybrid membranes, and providing stabilization, hence expanding the range of potential applications and improving the efficiency of polymer materials as antibacterial agents [[101], [103]].

As discussed in this section, MOFs proved to have remarkable antibacterial performance through different mechanisms. Not only can MOFs exhibit excellent drug release properties controlled by their unique porous structure, but they also possess distinct properties for the release of therapeutic agents. However, MOFs typically show low bioactivity, a property characteristic to BGs. Consequently, integrating MOFs with BGs gains attention for using them in various composite systems, particularly in hydrogel matrices, as discussed in the previous section. This combination might take advantage of the individual and cumulative properties of MOFs, BGs and hydrogels, leading to better physicochemical and biological properties. A comprehensive analysis of the most recent MOFs/BGs studies revealed increasing research interest focused on investigating and developing MOFS/BGs composite systems, emphasizing their potential to advance biomedical research through improving available therapeutic strategies. In this context, Table 1 presents a summary of the state-of-the art studies in this field.

## 6 Conclusion

MOFs and BGs have recently gained remarkable popularity in the biomedical field due to their unique and complementary properties. Hence, the first part of this review was focused on highlighting the similarities and differences between MOFs and BGs, followed by a detailed analysis of their synergistic combination. Examples from the literature indicated that by using MOFs/BGs composites not only superior mechanical properties and controlled degradation rates, but also improved biocompatibility, can be achieved. All these properties make MOFs/BGs combinations suitable for a wide range of applications, including bone regeneration, wound healing, and infection control. Furthermore, the incorporation of MOFs and BGs into hydrogels expanded their application potential, leading to the development of advanced platforms for targeted drug delivery and soft tissue engineering. Lastly, this review presented the antibacterial mechanisms of MOFs, which primarily arise from the ability of the MOFs to release both ions and organic ligands. These antibacterial properties can become even stronger if MOFs are used simultaneously and synergistically with BGs, obtaining in this way composite materials which can prevent the growth of bacteria and impede the formation of biofilms. Hence, BGs/MOFs composites represent an innovative solution for addressing the critical challenges associated with infections after implantation, especially for tackling highly resistant bacterial infections, for example when used as coating of metal implants. The ultimate goal is to obtain multifunctional systems that can target various biological processes, closely mimicking the structure and functions of native tissues. Thus, important advancements in the field of regenerative medicine and implantable devices can be achieved.

Future research directions should be focused on optimizing the synthesis and integration methods of MOFs/BGs composites to improve their functional properties, considering also the time dependent ion release kinetics. This can be achieved by modifying the synthesis route, leading to new compositions, degradation profiles and porosity, further enabling the control of ion release kinetics. Moreover, in the realm of developing a new generation of biomedical devices, the integration of MOFs and BGs with other biomaterials and advanced fabrication technologies, such as 3D printing, should be investigated. In this way, complex structures mimicking the hierarchical architecture of the tissue can be fabricated.

Because most of the existing studies are mainly focused on using MOFs/BGs composites in bone regeneration therapies, efforts should also be made towards translating these composites to soft tissue engineering. In this regard, efforts should be made to adjust the material composition and physicochemical properties to make the transition to soft tissue applications. Furthermore, to maximize the therapeutic potential of MOFs/BGs, it is crucial to gain a deep understanding of the interactions between BGs, MOFs and cells, including their immunomodulatory potential, making in this way possible to develop novel bioactive materials that target and modulate specific cellular pathways. By carrying out in-depth studies on cell-material interactions and immune response, valuable insights about cellular mechanisms can be gained. Even if the technology is still in its infancy, the ongoing advancements regarding MOFs/BGs composites underscore their potential as innovative functional systems which could offer another perspective for the design and application of biomaterials in medicine, leading to advanced medical therapy and improving the quality of life of patients. However, the first step towards bringing these materials to clinical use consists of successful in vitro and in vivo preclinical testing, followed by regulatory approval.

## CRediT authorship contribution statement

**Andrada-Ioana Damian-Buda:** Writing – review & editing, Writing – original draft, Conceptualization. **Nariman Alipanah:** Writing – review & editing. **Faina Bider:** Writing – original draft, Conceptualization. **Orhan Sisman:** Writing – review & editing. **Zuzana Neščáková:** Writing – review & editing, Funding acquisition, Conceptualization. **Aldo R. Boccaccini:** Writing – review & editing, Writing – original draft, Supervision, Funding acquisition, Conceptualization.

## Declaration of competing interest

The authors declare that they have no known competing financial interests or personal relationships that could have appeared to influence the work reported in this paper.

## Data Availability

No data was used for the research described in the article.
